# Mechanisms by which spinal cord stimulation intervenes in atrial fibrillation: The involvement of the endothelin-1 and nerve growth factor/p75NTR pathways

**DOI:** 10.1515/med-2023-0802

**Published:** 2023-10-05

**Authors:** Yiyan Peng, Peng Li, Wei Hu, Qi Shao, Panpan Li, Haiyue Wen

**Affiliations:** Xiaogan Central Hospital Postgraduate Training Base of Jinzhou Medical University, Xiaogan, 432100, Hubei, China; Xiaogan Hospital Affiliated to Wuhan University of Science and Technology, No. 6, Square Street, Xiaonan District, Xiaogan, 432100, Hubei, China; Xiaogan Central Hospital, Xiaogan, 432100, Hubei, China; Hubei Province Key Laboratory of Occupational Hazard Identification and Control, Wuhan University of Science and Technology, Wuhan, 430065, China; Xiaogan Hospital Affiliated to Wuhan University of Science and Technology, Xiaogan, 432100, Hubei, China; Jinzhou Medical University, Jinzhou, 121001, China

**Keywords:** atrial fibrillation, spinal cord stimulation, autonomic nerves, nerve growth factor, endothelin-1, NF-kB p65, p75NTR

## Abstract

Can the spinal cord stimulation (SCS) regulate the autonomic nerves through the endothelin-1 (ET-1) and nerve growth factor (NGF)/p75NTR pathways and thus inhibit the occurrence of atrial fibrillation (AF)? In our research, 16 beagles were randomly divided into a rapid atrial pacing (RAP) group (*n* = 8) and a RAP + SCS group (*n* = 8), and the effective refractory period (ERP), ERP dispersion, AF induction rate, and AF vulnerability window (WOV) at baseline, 6 h of RAP, 6 h of RAP + SCS were measured. The atrial tissue was then taken for immunohistochemical analysis to determine the localization of ET-1, NGF, p75NTR, NF-kB p65, and other genes. Our results showed that SCS attenuated the shortening of ERP in all parts caused by RAP, and after 6 h of SCS, the probability of AF in dogs was reduced compared with that in the RAP group. Moreover, the expression of ET-1, NGF, and p75NTR in the atrial tissues of dogs in the RAP + SCS group was significantly increased, but the expression of NF-kB p65 was reduced. In conclusion, SCS promotes the positive remodeling of cardiac autonomic nerves by weakening NFκB p65-dependent pathways to interfere with the ET-1 and NGF/p75NTR pathways to resist the original negative remodeling and inhibit the occurrence of AF.

## 1 Introduction

Atrial fibrillation (AF) is the most common arrhythmia, and its basic pathophysiology includes electrical remodeling, structural reconstruction, and autonomic remodeling [1]. Previous studies have demonstrated that spinal nerve stimulation may inhibit rapid atrial pacing (RAP)-induced AF by inhibiting autonomic remodeling, and the mechanism of its action may be related to the expression of nerve growth factor (NGF) [2]. Many studies have found that cardiac autonomic nerve growth is closely related to NGF, and endothelin-1 (ET-1) can promote the synthesis of NGF by cardiomyocytes, thereby causing cardiac sympathetic abnormalities and altering cardiac autonomic imbalance [3,4,5], but the exact signaling pathway is unknown. This study aimed to use the spinal cord stimulation (SCS) of an AF dog model to determine whether the ET-1 and NGF/p75NTR pathways can promote the positive remodeling of cardiac autonomic nerves and affect the changes in atrial myocyte channel proteins, thereby changing the electrophysiological characteristics of the myocardium and promoting positive electrical remodeling of atrial muscle, so as to antagonize the negative effects of antigens, intervene in the triggering and maintenance of AF, and thus treat AF.

## 2 Materials and methods

Sixteen beagles ranging from 15 to 20 kg were used in this experiment. All procedures were performed under 3% sodium pentobarbital anesthesia with an initial dose of 1 mL/kg and a maintenance dose of 2 mL/h. The depth of anesthesia was monitored throughout the experiment by examining heart rate, respiratory rate, and toe crush responses. All dogs were endotracheally intubated and ventilated on a ventilator (MAO01746, Harvard Apparatus, Holliston, USA). A catheter was introduced into the left femoral artery to monitor the systemic arterial pressure, and the body surface electrocardiogram and blood pressure were recorded throughout the experiment with a computer laboratory system (Lead 2000B, Jingjiang Inc., Wuhan, China). Intravenous fluids were injected into the left femoral vein to maintain fluid loss. Dogs maintained the body nuclear temperature at 36.5 ± 1.5°C with open thoracotomy in the left and right thoracic fourth intercostal space.

### 2.1 Program stimulation

**Figure 1:**
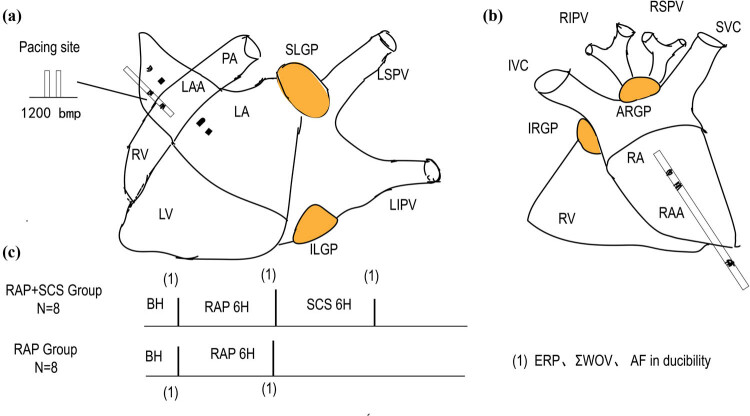
Schematic diagram of electrodes (a and b) and flow chart of experiments (c). AF = atrial fibrillation; ARGP = the right anterior ganglionic plexus; BH = baseline; ERP = effective refractory period; ILGP = the left lower ganglionic plexus; IRGP = what was found in the lower right ganglionic plexus; IVC = inferior vena cava; LA = left atrium; LAA = left atrial appendage; LIPV = left upper pulmonary vein left ventricle; PA = pulmonary artery; RA = right atrium; RAA = right atrial appendage; RIPV = the right lower pulmonary vein; RSPV = the right upper pulmonary vein; RV = right ventricle; SCS = spinal cord stimulation; SLGP = the left upper ganglionic plexus; SVC = superior vena cava; RAP = rapid atrial pacing; WOV = window of vulnerability; ΣWOV = cumulative WOV.

Acute atrial remodeling induced by 6 h RAP was performed at 1,200 beats/min (2 thresholds) in the left atrial appendage. The effective refractory period (ERP) in the atrial and pulmonary veins was determined by reducing S1–S2 with programmed stimulation of S1 = 330 ms, a 10-diastolic threshold. The S1–S2 interval was reduced by 10 ms from the initial 180 ms, followed by a 2 ms reduction when near the ERP. The window of vulnerability (WOV), a measure of the propensity for AF inducibility, was determined by the longest coupling interval of the premature beat (S1–S2) minus the shortest S1–S2, which induced AF. The cumulative WOV was the sum of WOVs at all sites in each dog [6,7,8]. AF was induced by the S1S1 (a procedure of 120, 100, and 75 ms cycle length for 5 s each and repeated three times for each frequency) (Figure 1). AF was defined as an irregular atrial rate >500 bpm lasting >5 s [9].

### 2.2 SCS

A small incision was made in the dorsal thoracic spine (T1–T2 level), and the thoracic epidural cavity was punctured with a Tuohy needle until the loss of resistance. The electrode was then introduced into the epidural cavity through this cannula, with the electrode tip oriented to the level of the T1–T2 spinal cord, slightly to the left of the midline. The end of the electrode was connected to a stimulator (S88, Grass Instruments, Quincy, MA) and to generate 50 Hz pulses with a 0.2 ms duration. Continuous stimulation occurred for 6 h.

### 2.3 Histological staining

At the end of the experiment, the atrial tissue was rapidly excised and fixed in 4% paraformaldehyde at room temperature. Paraffin-embedded tissue was cut into 5 μm sections. Immunofluorescence staining was used to determine the expression and localization of ET-1, NGF, p75NTR, NF-κB p65, and tyrosine hydroxylase (TH) in tissues. Sections were incubated in PBS containing 10% FBS for 60 min and incubated overnight with the primary antibody at 4°C, including anti-ET-1 (ABclonal, China), anti-NGF (Abcam, Cambridge, UK), anti-p75ntr (Abcam, Cambridge, UK), anti-NF-κB p65 (Abcam, Cambridge, UK), and anti-TH (Abcam, Cambridge, UK). Sections were washed with PBS and incubated with secondary antibodies for 1 h at 37°C. After the sections were washed, they were visualized using the DAB reagent. Hematoxylin was counterstained with cores dehydrated with ethanol and sealed with glycerol gelatin. Blinded analysis was performed using Image-Pro Plus 6.0 (Media Cybernetics).

### 2.4 mRNA analysis

Total RNA was obtained from atrial tissue using TRIzol reagent (Servicebio) and then reverse transcribed to cDNA. Finally, the PCR assay was performed. The primers are shown in Table 1. The relative expression level was calculated using the 2^−ΔΔCt^ method.

**Table 1:** Primer sequences of the genes that were verified by RT-PCR

Primer name	Forward sequence (5′–3′)	Reverse sequence (5′–3′)
ET-1	CTGCTCCTGCTCTTCCCTGAT	TGTGGTCTGTTGCCTTTGTGAT
TrkA	GCTGTCTTTGCCTGCCTCTT	GACAAGGAACTGCCACCTAATG
NGF	TCCTTCCTGGGCATGGAATC	ACAGCACTGTGTTGGCATAGA
P75NTR	TGGACAGCGTGACGTTCTCC	GATCTCCTCGCACTCGGCGT
NF-KB p65	GTGCAGAAAGAAGACATTGA	AGGCTAGGGTCAGCGTATGG

### 2.5 Experimental scheme

The 16 beagles were randomly divided into two groups, one for 6 hours of rapid atrial pacing (*n* = 8) and the other group for 6 hours of rapid atrial pacing followed by 6 hours of spinal cord stimulation in the T1–T2 spinal cord level (delivered at 50 Hz, 0.1 ms pulse width, by approximately 90% of the motion threshold) (*n* = 8). Atrial electrophysiological parameters (ERP, ERP dispersion, and WOV) were measured at three time points: baseline, 6-h RAP, and 6-h RAP + 6-h SCS. Finally, the removed atrial tissue was used for protein blotting and messenger RNA (mRNA) analysis.

### 2.6 Data analysis

Measurement data of the study were expressed using the mean ± standard deviation and analyzed by paired *t* tests, with *P* < 0.05 considered statistically significant. Data analysis and plotting were performed using the Graphpad Prism software.

## 3 Results

### 3.1 Effect of SCS on ERP, ΣWOV, and AF inducible properties

**Figure 2:**
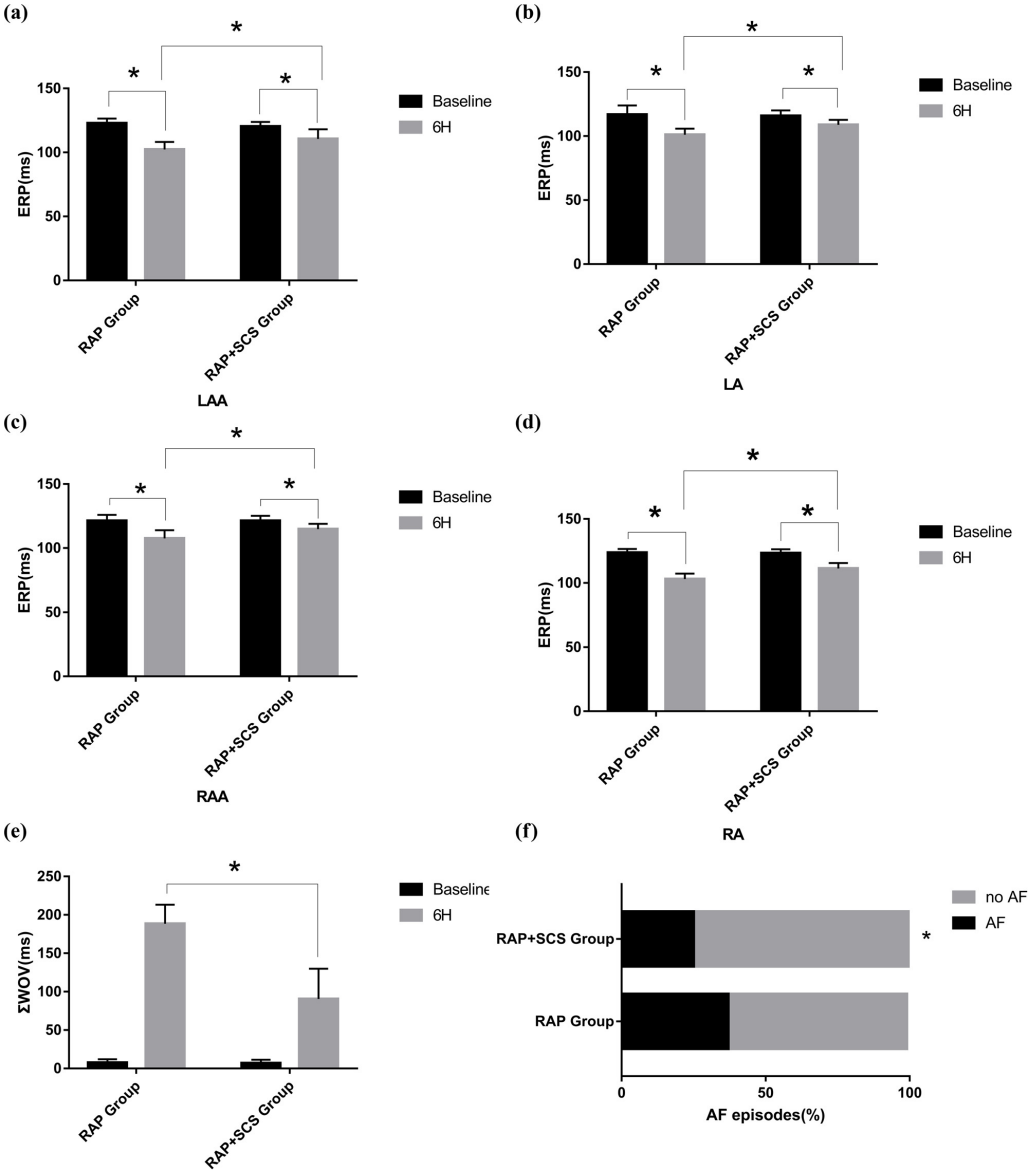
Effect of SCS on ERP (a–d), ΣWOV (e), and AF episodes (f) at all sites of the atrium. *P < 0.05.

In Figure 2, at the baseline, ERP was measured at any tissue site. SCS attenuated the shortening of the ERP at all sites caused by the RAP (Figure 2a–d). SCS caused an increase in ΣWOV (Figure 2e). After 6 h of RAP, AF was observed in three of the eight dogs in the RAP group and two of the eight dogs in the SCS + RAP group with AF (37.5% vs 25%, *P* > 0.05) (Figure 2f) (*P* < 0.05 compared with RAP group).

### 3.2 Effect of SCS on the molecules associated with the ET-1 signaling pathway

#### 3.2.1 Localization and expression of ET-1, NGF, p75NTR, NF-κB p65, and TH in atrial tissue

**Figure 3:**
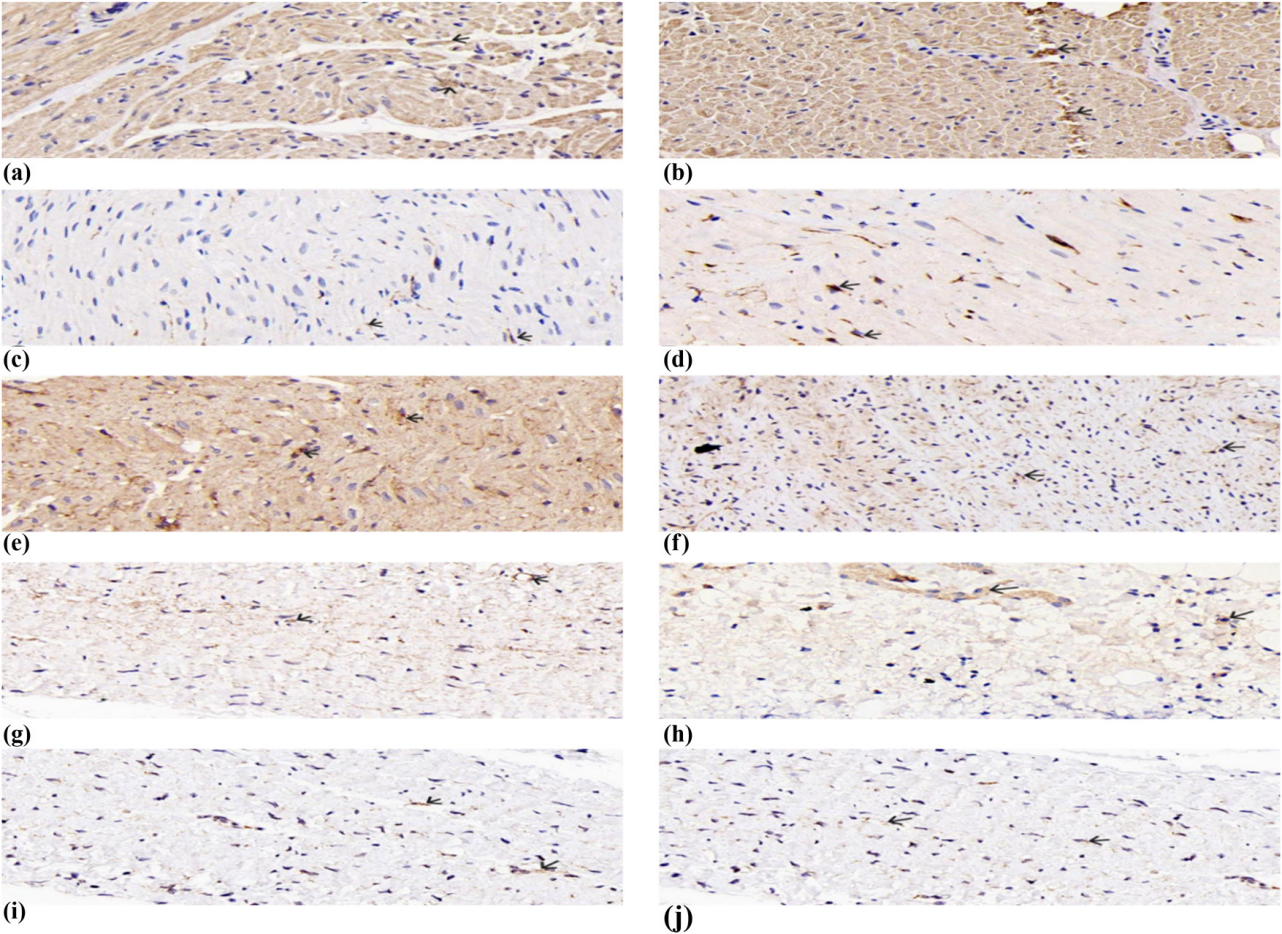
Localization of ET-1 (a and b), NGF (c and d), p75NTR (e and f), NF-κB p65 (g and h), and TH (i and j) in the atrial tissue was determined by immunohistochemistry. Scale bar represents 50 μm.

**Figure 4:**
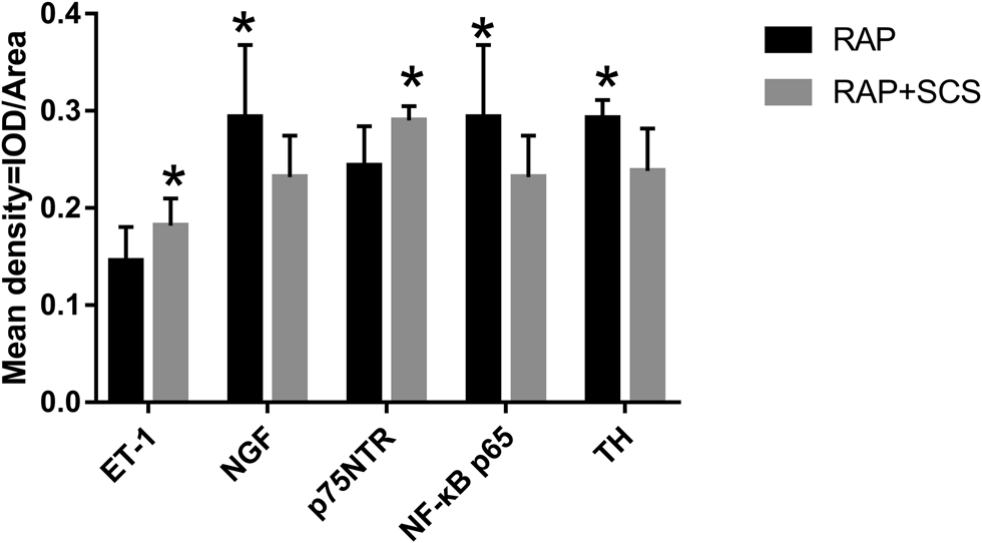
Average optical densities of the various target proteins in the atrial tissue. *P < 0.05.

The localization of ET-1, NGF, p75NTR, NF-κB p65, and TH in atrial tissue was determined by immunohistochemistry (Figure 3). Atrial tissue expression of ET-1 (Figure 3a and b), p75NTR (Figure 3c and d), NF-κB p65 (Figure 3e and f), NGF (Figure 3g and h), and TH (Figure 3i and j) in the RAP + SCS and RAP groups was found. The scale bar represents 50 μm. The figure shows the average optical density (mean density) data for various target proteins expressed as mean ± SD (Figure 4). The expression of ET-1, NGF, and p75NTR was higher in the RAP + SCS group than in the RAP group, and NF-κB p65 and TH expressions were lower than those in the RAP group. *P < 0.05.

#### 3.2.2 mRNA expression of ET-1, NGF, TrkA, p75NTR, and NF-κB p65

**Figure 5:**
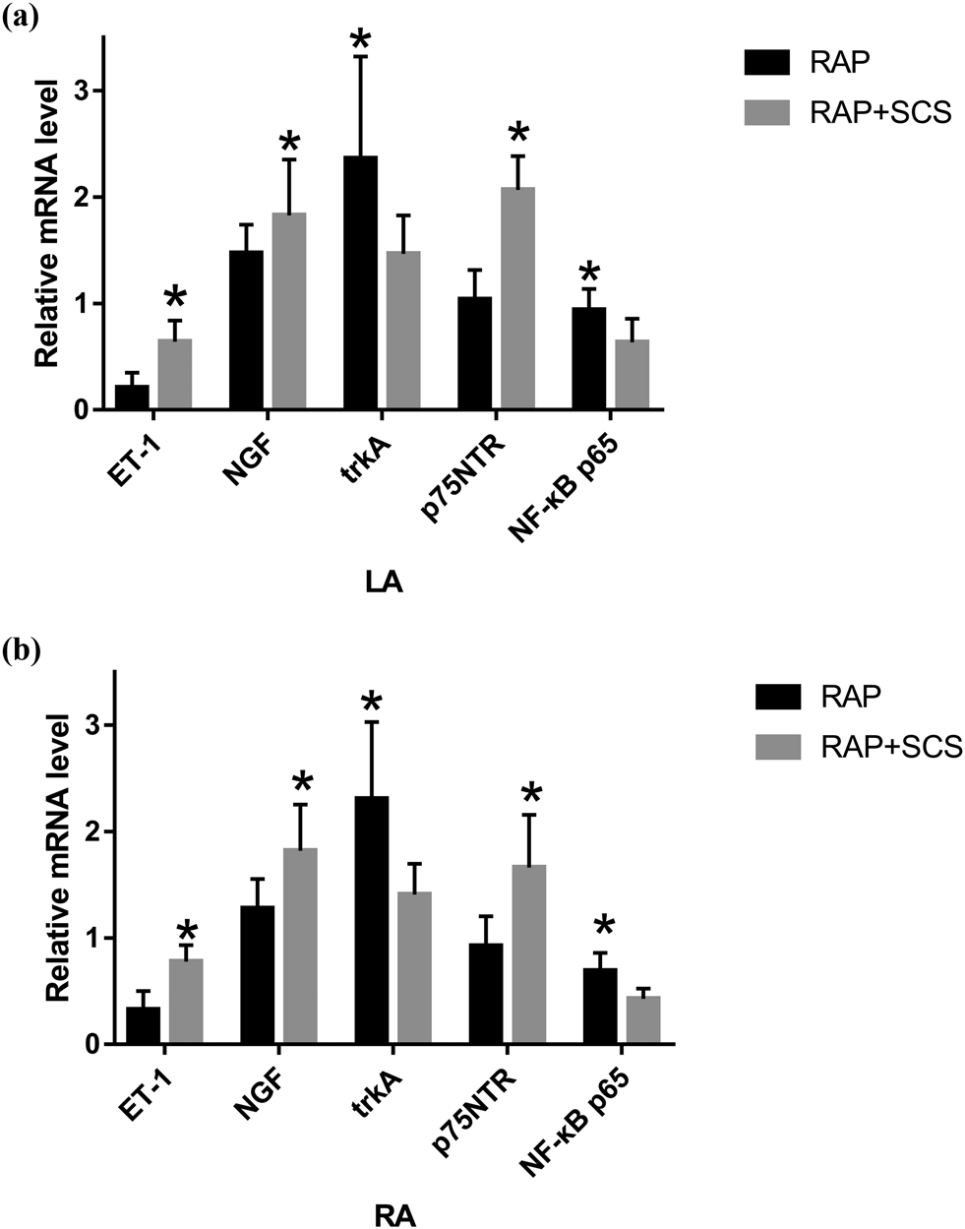
mRNA levels of ET-1, NGF, p75NTR, NF-κB p65, and trkA, respectively, in the left atrium tissue (a) and in the atrium tissue (b). *P < 0.05.

As shown in left atrium (Figure 5a) and right atrium (Figure 5b), the mRNA expression of ET-1, NGF, and p75NTR was higher in the RAP + SCS group than in the RAP group (*P* < 0.05), and the mRNA expression of NF-κB p65 and trkA was lower in the RAP + SCS group than in the RAP group (*P* < 0.05).

## 4 Discussion

Autonomic nervous system (ANS) imbalance is one of the important mechanisms by which atrial arrhythmias occur [10]. Although pulmonary venous isolation is widely used for AF ablation, it is an invasive procedure with potentially serious complications. SCS is the delivery of electrical stimulation to spinal cord segments through implanted electrodes to treat a variety of painful conditions, including chronic back pain and intractable angina [11,12]. Recent studies have shown that SCS can be used to prevent the development of AF after cardiac surgery [13]. We controlled the development of AF by using a spinal cord-stimulating canine animal model to modulate the ANS imbalance. Previous studies have demonstrated that SCS has a direct effect on cardiac electrophysiology [2,14]. This study also confirmed that SCS breaks the vicious cycle of autonomic electrical remodeling and structural remodeling by inhibiting the shortening of atrial ERP and the expansion of WOV, thereby inhibiting negative autonomic remodeling and inhibiting the occurrence of AF.

ET-1 is an endogenous vasoconstrictor peptide, and local ET-1 production in the heart is secreted by the endocardium, myocardium, and coronary artery endothelium thereby acting on cardiomyocytes in paracrine and autocrine ways [15,16]. Previous studies have suggested that ET-1 may play a key role in the regulation of sympathetic activity and is associated with the development of sympathetic neurons [17,18,19,20]. NGF is a 118-amino acid glycoprotein composed of three subunits (α, β, and γ complexes), and β-NGF is responsible for its biological activity. NGF is associated with sympathetic distribution, and its increase triggers nerve germination in noninfarcted ventricles and atria [21]. Sympathetic overgermination may be an important factor in sympathetic remodeling leading to arrhythmias [22]. Elevated NGF levels and excessive sympathetic innervation lead to arrhythmias, including AF [5]. ET-1 has been shown to increase (NGF, mRNA, and protein levels during the development and regeneration of cardiac sympathetic innervation [5,23]. In this study, we found that the mRNA and protein expression of ET-1 and NGF in the atrial tissue of beagle dogs with SCS RAP for 6 h was elevated, indicating that SCS can activate the ET-1 and NGF signaling pathways.

The receptors of cardiac NGF are divided into the high affinity receptor TrkA and the low affinity tumor necrosis factor receptor P75NTR [24], which maintain cardiac sympathetic neuron growth and are closely related to stimulating axon regeneration. NGF/TrkA signaling plays an important role in enhancing normal cardiac calcium circulation and the normal function of the cardiovascular system [25,26]. Studies have demonstrated that NGF/TrKA signaling is associated with the development of AF [27]. In this study, the expression of p75NTR in the SCS group was higher than that in the RAP group, and the TrkA expression was lower than that in the RAP group, indicating that NGF in the SCS group mainly binds to p75NTR. Therefore, we speculate that the activation of ET-1 and NGF/p75NTR is closely related to the regulation of cardiac autonomic remodeling by SCS.

Nuclear factor kappa B (NF-κB) is a family of dimer transcription factors, and the NF-κB transcription factor system plays various roles in nervous system development and postnatal physiological processes [28]. Nuclear factor-κB p65 (NF-κB p65) activation can upregulate cardiac NGF and promote sympathetic innervation [29,30]. This study showed that the expression of NF-κB p65 was reduced in the SCS group, demonstrating that the activation of NF-κB p65 was inhibited, thus inhibiting the sympathetic nerve growth. The present study also showed that TH expression was significantly reduced in the SCS group, further confirming that sympathetic nerve growth was inhibited. All these results indicate that the ET-1 and NGF/p75NTR signaling pathways in the SCS can attenuate the sympathetic innervation through the NF-κB p65-dependent pathway and promote positive remodeling of the cardiac autonomic nerve to resist the original negative remodeling and inhibit the occurrence of AF.

### 4.1 Clinical significance of this study

This study further explored the mechanism of ET-1 and NGF/p75NTR-dependent pathways in SCS in AF, providing more beneficial effects for future SCS in regulating the cardiac ANS.

### 4.2 Research restrictions

This study has some limitations. First, this experiment was performed under general anesthesia. Based on previous research, plasma pentobarbital concentration in this experiment speeded up and slowed down early repolarization and reduce the amplitude of the action potential [31,32,33]. In the future, we hope to design experiments at anesthetic concentrations that do not affect changes in dog electrophysiology. Second, the expression levels of cardiac molecular substances in normal animals (beagle dogs not receiving RAP) were not measured in this study. To provide a comprehensive comparison, we plan to measure the expression levels of cardiac molecular substances in normal animals (beagle dogs not receiving RAP).
